# Homozygous Co-Deletion of Type I Interferons and *CDKN2A* Genes in Thoracic Cancers: Potential Consequences for Therapy

**DOI:** 10.3389/fonc.2021.695770

**Published:** 2021-06-24

**Authors:** Marion Grard, Camille Chatelain, Tiphaine Delaunay, Elvire Pons-Tostivint, Jaafar Bennouna, Jean-François Fonteneau

**Affiliations:** ^1^ Université de Nantes, Inserm, CRCINA, Nantes, France; ^2^ Labex IGO, Immunology Graft Oncology, Nantes, France; ^3^ CHU de Nantes, oncologie thoracique et digestive, Université de Nantes, Nantes, France

**Keywords:** lung cancer, mesothelioma, type I interferon, CDKN2A (p16), homozygous deletion, immunotherapy, STING

## Abstract

Homozygous deletion (HD) of the tumor suppressor gene *CDKN2A* is the most frequent genetic alteration in malignant pleural mesothelioma and is also frequent in non-small cell lung cancers. This HD is often accompanied by the HD of the type I interferons (IFN I) genes that are located closed to the *CDKN2A* gene on the p21.3 region of chromosome 9. IFN I genes encode sixteen cytokines (IFN-α, IFN-β…) that are implicated in cellular antiviral and antitumor defense and in the induction of the immune response. In this review, we discuss the potential influence of IFN I genes HD on thoracic cancers therapy and speak in favor of better taking these HD into account in patients monitoring.

## Frequent Homozygous Co-Deletion of the *CDKN2A* Tumor Suppressor Gene and the IFN I Genes in Thoracic Cancers

Non-small cell lung cancer (NSCLC) is the most common cause of cancer death worldwide often due to long-term tobacco smoking. Malignant pleural mesothelioma (MPM) is a rare cancer that is mainly due to asbestos exposure. As other cancers, some genomic alterations are found in NSCLC and MPM tumor cells, especially in locus containing tumor suppressor genes. These alterations are in part responsible for the disease.

**Figure 1:**
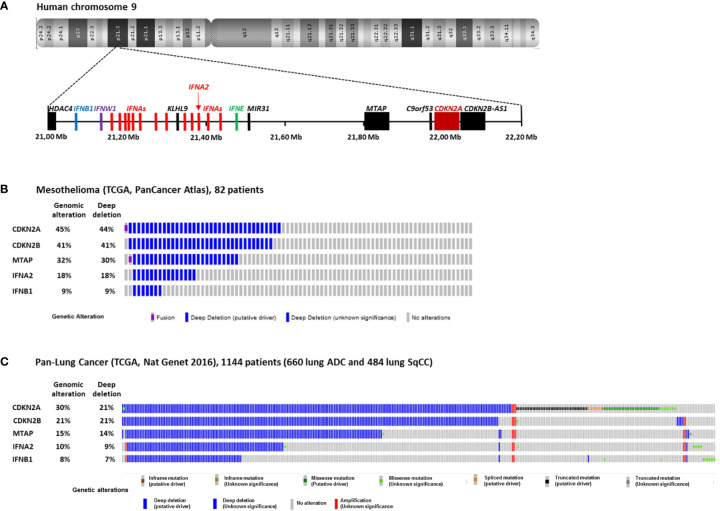
Homozygous Deletions in the p21.3 region of chromosome 9 in MPM and NSCLC. **(A)** Schematic representation of genes present in the p21.3 region of chromosome 9 between positions 21,000,000 and 22,200,000 drawn from UCSC Genome Browser (https://genome.ucsc.edu/). **(B)** Oncoprint representation of *CDKN2A*, *CDKN2B*, *MTAP*, *IFNA2* and *IFNB1* genomic alterations found in tumor samples of 82 MPM patients. Oncoprint was performed with cbioportals website (http://www.cbioportal.org/) using TCGA Pancancer atlas data. **(C)** Oncoprint representation of *CDKN2A*, *CDKN2B*, *MTAP*, *IFNA2* and *IFNB1* genomic alterations found in tumor samples of 1144 NSCLC patients. Oncoprint was performed with cbioportals website (http://www.cbioportal.org/) using TCGA Pan lung cancer data. Only the patients with at least one genomic alteration in the five genes are shown. ADC, adenocarcinoma; SqCC, squamous cell carcinoma.

In MPM cells, the most frequent genomic alteration is the homologous deletion (HD) in the p21.3 region of chromosome 9 (1–6). These HDs are variable in length but they mainly overlap at the level of the cyclin-dependent kinase inhibitor 2A (*CDKN2A*) tumor suppressor gene located in this region (**Figures 1A, B**). Fluorescence *in situ* hybridization (FISH) studies reported that *CDKN2A* gene HDs are found in 60 to 80% of patients (3–6). Copy number alteration study from The Cancer Genome Atlas (TCGA) reported a lower frequency of 44% of patients with *CDKN2A* gene HD in MPM (**Figure 1B**) (7). However, TCGA study is performed on tumor biopsies that often contain non-malignant cells. These non-malignant cells may mask *CDKN2A* gene HD that are only present in tumor cells. Thus, some patients with *CDKN2A* gene HD were probably not detected in the TCGA study.

In NSCLC, *CDKN2A* gene HDs were also identified in the 1990s (8–10). They were then found more frequently in a subset of patient with intact retinoblastoma (rb) pathway (11). FISH studies on 85 and 19 NSCLC patients reported *CDKN2A* gene HD in 21 and 29% of patients respectively (12, 13), and FISH study on 31 squamous cell carcinoma (SqCC) patients reported them in 16% (14). TCGA study on 1144 NSCLC patients (660 lung adenocarcinoma and 484 lung SqCC) reported *CDKN2A* gene HD in 21% of patients (**Figure 1C**) (15).

The *CDKN2A* gene encodes several proteins, notably p16^INK4a^ and p14^arf^ that are implicated in the regulation of the cell cycle. p16^INK4a^ binds to cyclin-dependent kinase 4 and 6 (CDK4/6) and inhibits its capacity with cyclin D1 to phosphorylate rb protein and the translocation of the transcription factor E2F from the cytoplasm to the nucleus (16). In absence of p16^INK4a^, E2F translocates to the nucleus and allows the transition from G1 phase to S phase of the cell cycle. p14^arf^ also acts as a tumor suppressor *via* the p53 pathway and its absence favors the entry in the cell cycle.

Close to *CDKN2A* gene, *CDKN2B* and *MTAP* are two other genes that are often co-deleted with *CDKN2A* in MPM (**Figure 1B**) and NSCLC (**Figure 1C**). *CDKN2B* encode the p15^Ink4b^ protein that interacts with CDK4/6 and inhibits its activation by cyclin D and thus acts as a tumor suppressor (17). *MTAP* encodes the S-methyl-5’-thioadenosine phosphorylase (MTAP) implicated in the polyamine metabolism (18).

Further downstream from *CDKN2A* and *MTAP* in the p21.3 region of human chromosome 9, a cluster of 16 genes encodes the type I interferons (IFN I): IFN-β, IFN-ε, IFN-ω and 13 IFN-α (**Figure 1A**) (19). *IFNB1* is the furthest gene from *CDKN2A*. In the 1990s, IFN I genes HDs were identified in a fraction of NSCLC and MPM patients with *CDKN2A* gene HD (1, 2, 8, 20). We recently reported that in 78 short-term–cultured MPM cell lines, 57 (73%) and 18 (23%) cell lines harbors *CDKN2A* and *IFNBI* genes HD respectively, whereas in TCGA study performed on 82 patients, these percentage where smaller, probably due to non-malignant cells contamination (44 and 9%) (**Figure 1B**) (21). Thus, about 10 to 20% of mesothelioma patients present HD of all the IFN I genes. In NSCLC, the TCGA study on 1,144 patients reported 21 and 7% of patients with *CDKN2A* and *IFNBI* gene HDs respectively (**Figure 1C**). Interestingly, NSCLC patients with IFN I and *CDKN2A* gene HDs have a significantly worst disease free survival than patients with only *CDKN2A* gene HD (22), suggesting a tumor suppressor role for IFN I genes in this cancer.

## The Type I Interferon Response in Thoracic Cancers

**Figure 2:**
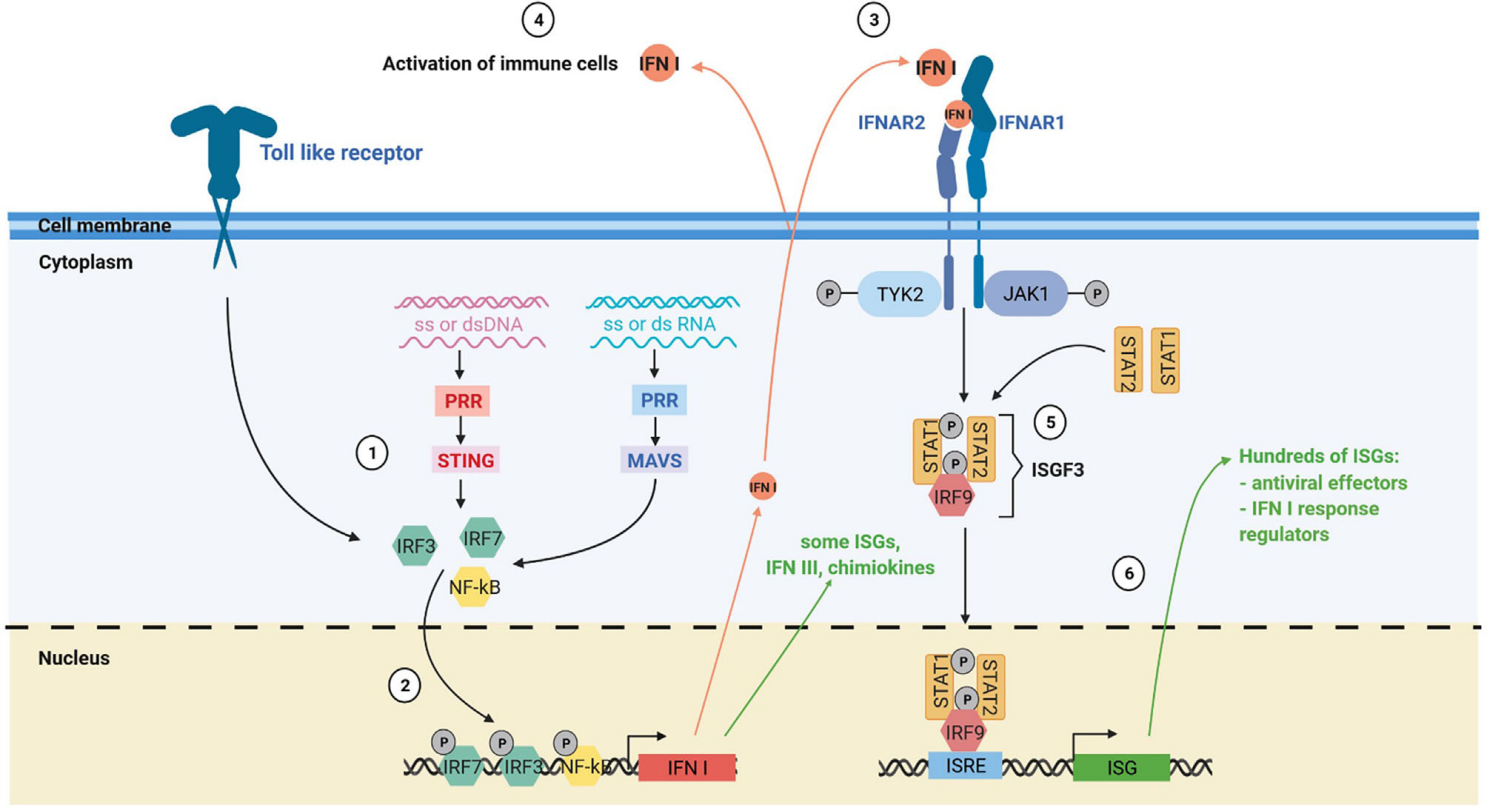
The IFN I response. (1) The IFN I response is triggered by different stimuli such as ssDNA, dsDNA, ssRNA and dsRNA *via* the toll like receptors, the MAVS and the STING pathway. (2) Activation of these pathways induces the nuclear translocation of transcription factors such as IRF3, IRF7 and NF-kB that trigger IFN I production and expression of some interferon stimulated genes (ISGs). (3) Secreted IFN I trigger IFNAR signaling on neighboring cells, notably immune cells. (4) IFN I and other soluble factors of the IFN I response activate the immune response. (5) Activation of IFNAR signaling by IFN I leads to the formation of the interferon stimulated gene factor 3 complex. (6) This complex translocates to the nucleus and activates numerous ISGs with antiviral, immunomodulatory and regulatory functions. PRR, pattern recognition receptors.

Type I interferon (IFN I) response is key in antiviral immune response (**Figure 2**). The IFN I response allows infected and immune cells to report *via* IFN-α and -β secretion the presence of the virus to neighboring cells and to the immune system *via* the IFN-α/-β receptor (IFNAR) which is expressed by virtually all somatic cells (23). The presence of viral genome or intermediaries of its replication is detected by cytoplasmic pattern recognition receptors (PRR) and lead to the production of IFN I *via* two main pathways: the stimulator of interferon genes protein (STING) pathway for DNA viruses and the mitochondrial antiviral-signaling protein (MAVS) pathway for RNA viruses (23, 24). IFN I are also produced by immune cells notably *via* Toll like receptor (TLR) activation, especially plasmacytoid dendritic cells (pDC) (25). IFN-β is expressed by all nucleated cells in response to infection, whereas the IFN-α are mainly produced by immune cells. Among IFN-α, IFN-α2 was the first cytokines to be approved in clinics for cancer treatment in 1986 and is the most studied (26).

Cells exposed to IFN I express hundreds of IFN-stimulated genes (ISGs). Many ISGs encode proteins that induce a state of anti-viral resistance. These antiviral proteins act by blocking the different stages of the viral cycle, from the entry of the virus, through the inhibition of its replication, to the release of its progeny by the infected cell (23, 27). The IFN I also play a crucial role in the induction and the regulation of the antiviral adaptive immune response, notably by favoring antigen cross-priming by dendritic cells (28, 29). However, during chronic infection, prolonged IFN I response can have deleterious effects by inducing immune dysfunctions (30).

IFN I response is often induced during cancer development and treatments. Several pathways are involved in this induction. Presence of mitochondrial or nuclear DNA in the cytoplasm of tumor cells can induce the secretion of IFN I *via* the STING pathway (31–39). Expression of endogenous retrovirus (ERV) under the form of dsRNA due to epigenetic deregulation in tumor cells can also trigger the expression of IFN I *via* the MAVS pathway (35, 40–44). Non-malignant cells from the tumor microenvironment, such as phagocytic cells notably dendritic cells (DC), can also produce IFN I *via* activation of the STING pathway after engulfment of dead tumor cells. This occurs due to accumulation of DNA from engulfed dead tumor cells in DC cytoplasm (39, 45–47). A recent analysis of 31 cancer types in TCGA database by Liu et al. shows that, lung adenocarcinoma, MPM and SqCC are the 3rd, 6th and 8th respectively in the intensity of an IFN I signature based on the expression of 38 ISGs (35). Globally, IFN I signature correlates with the degree of immune cells infiltration, but some tumors with an interferon signature and with no immune cells infiltration were also found.

IFN I signaling is able to modulate the expressions of hundreds of genes (27). This signaling pathway also induces expression of noncoding RNAs including long noncoding RNAs, miRNA and ERV RNA (27, 41, 48). Due to its potential toxicity and inflammatory effects, IFN I production and signaling are tightly regulated by numerous positive and negative regulators, many of which are ISGs (49). Thus, induction of IFN I response in tumors has multiple complex effects that are rather unfavorable to tumor development. The IFN I can restrict tumor growth by reducing proliferation of tumor cells, inducing their apoptosis, limiting their migratory capacity and inhibiting angiogenesis (50, 51). Furthermore, they increase antigen presentation by HLA molecules, stimulate the innate and adaptive antitumor immune response and inhibit CD4+ regulatory T cells (30, 50, 52–56). IFN I were shown to play an important role in tumor immuno-editing in mouse models of chemically-induced and transposable tumors (57). They signal tumor cells to the immune system. They are necessary for the priming of anti-tumor T cell response as the abrogation of IFN I signaling in CD8+ dendritic cells blocks their capacity to cross-present antigens in mouse (54, 55). They also participate in the activation of anti-tumor NK cell response (56). Induction of anti-tumor NK and T cell responses lead to the secretions of the type II interferon-gamma (IFN-γ) that will further shapes the immunogenicity and the immunosuppressive microenvironment of the tumor with the IFN I (58). Indeed, IFN I are also involved in setting up the immunosuppressive tumor environment by inducing the expression of numerous inhibitory molecules such as PD1 and PDL1 that block CD8+ T cell cytotoxicity (30, 50, 58). IFN I can also induce Indoleamine 2,3-dioxygenase (IDO) expression that reduces locally the amount of tryptophan needed for T cell functions and favors their differentiation in Treg (59). Thus, the IFN I in tumors play a dual role by stimulating the innate and adaptive immune response and inducing feedback mechanisms to control its magnitude.

IFN I in NSCLC modulates numerous pathways implicated in proliferation, survival and apoptosis of tumor cells including JAK/STAT, Src kinases, Vav proto-oncogene, PTEN/PI3-K/AKT, Crk proteins and MAP kinase signaling pathways (51). Several recent studies reported that a constitutive activation of the IFN I response in lung tumors correlates with tumor inflammation and immune checkpoint inhibitors efficacy (60–62). Furthermore, DNA damages and dysfunctions of the DNA damage response are inducers of the IFN I response and have been linked to immune checkpoint efficiency (37, 63). The renewed interest in IFN I response also comes from the observation that they are necessary for the radiotherapy abscopal response (64–67), and that they participate in the induction of the antitumor immune response by chemotherapy (68). This is also due to the identification of new potential immune checkpoint such as ADAR and Trex1 which are ISG that functions as negative regulators of the IFN I response by inactivating the nucleic acids that stimulate this response (35, 40, 65, 66). By blocking ADAR or Trex function, IFN I response is amplified which promotes the antitumor immune response. Thus, there is still a great interest to adapt treatments or find new therapeutic strategies to activate the IFN I response locally especially in cold tumors with no or low immune cells infiltrate.

## Potential Consequences of IFN I Genes HD for Thoracic Cancers Therapy

Given the central role of IFN I response in tumor immune surveillance, the frequent loss of all copies of IFN I genes that accompanied *CDKN2A* gene HD in tumor cells likely plays a role in tumor immune escape. In NSCLC, like in other cancers, patients with only *CDKN2A* gene HD have a longer survival compared to patients with IFN I and *CDKN2A* genes HD (22). Thus, IFN I genes act as tumor suppressors genes in malignant cells. Beside this study of Ye et al., nothing is known on the prognostic value and immunotherapy biomarker potential of IFN I genes HD in NSCLC, MPM and other cancers. These deletions were described as early as the mid-1990s and yet they have not been well documented. It may be because they were discovered in studies focusing on the *CDKN2A* tumor suppressor gene HD. Furthermore, reports that IFN I treatment in NSCLC and MPM has limited clinical benefit at that time, may have decrease the interest of studying IFN I gene HD. Finally, most studies on the role of IFN I on antitumor immune response were performed with IFNARko mouse models that are easier to obtain than mouse models ko for all IFN I genes. These IFNARko models are instrumental to understand the role of IFN I signaling on tumor cells and the different subtypes of immune cells but are less suitable to study the source of the IFN I production.

Questions arise regarding presence of IFN I genes HD in tumor cells. There are several potential cellular sources of IFN I secretion in tumors, that are basically tumor and immune cells. Thus, the first question is the role of IFN I production by tumor cells and if absence of this production is compensated by other cellular sources. Several recent studies suggest that triggering of the tumor cells IFN I production play a role in the induction of the anti-tumor immune response.

Kitajima et al. reported that the lack of response to immune checkpoint blockade (ICB) of patients with KRAS-LKB1–mutant lung cancers is due to the inhibition of STING expression *via* the loss of LKB1 (62). They show that KRAS-LKB1–mutant tumor cells are not able to sense cytoplasmic dsDNA *via* the STING pathway, and to produce IFN I in response. In consequence, T cells infiltration and PD-L1 expression in KRAS-LKB1-mutant tumors is reduced and ICB therapy is ineffective.

Demaria’s team showed that triggering of tumor cell IFN I response is necessary for induction of anti-tumor immune response by radiotherapy (64, 65). They first reported that abscopal response in mouse is abrogated when cancer cells in the irradiated tumor do not express cGAS/STING or overexpress the exonuclease Trex1 (65). Irradiation induces the presence of cytoplasmic DNA that triggers the IFN-β production *via* the STING pathway and lead to the expression of the ISG Trex1. This ISG is an exonuclease that degrades DNA in the cytoplasm and thus decreases IFN I response by tumor cells and triggering of the antitumor immune response. By inactivating Trex1 in tumor cells, the IFN I response induced by irradiation is increased, as well as the antitumor immune response. Thus, tumor cell IFN I response is essential for abscopal effect of radiation in that mouse model. Demaria’s team then showed that combination of radiotherapy and anti-CTLA4 blockade in NSCLC patients that have failed anti-CTLA4 alone or in combination with chemotherapy, induced IFN-β in the blood and an antitumor T cell response in responding patients (64).

These studies highlight the important role on the antitumor immune response of triggering the IFN I response *via* the STING pathway in tumor cells. However, IFN I response in tumor cells can also be induced by the sensing of endogenous dsRNA *via* the MAVS pathway and that also plays a role in the stimulation of the antitumor immune response (35, 40–44). Best evidences come from the study of an ISG, the adenosine deaminase acting on RNA (ADAR) that acts on the MAVS pathway like Trex1 does on the STING pathway (35, 40). The ADAR protein, by converting A to I, disrupts the normal A:U pairing which destabilizes the dsRNA into ssRNA. dsRNA edited by ADAR are no longer able to trigger the IFN I response by the dsRNA cytoplasmic sensor Mda5. Thus like Trex1, ADAR inactivates the stimuli at the origin of the IFN I production by tumor cells. In a mouse model, Ishizuka et al. reported that loss of *ADAR1* in tumor cells overcomes the resistance to immune checkpoint inhibitors by increasing the IFN I response *via* the MAVS pathway and, thus, the inflammation of the tumor microenvironment (40). This results was confirmed by Liu et al., that show that both, the MAVS and the STING pathway are needed to maintain the IFN I response in tumor cells that have lost ADAR (35).

Altogether these studies on the STING and the MAVS pathway show that triggering of the IFN I response in tumor cells is central to inflame the microenvironment. However, it does not establish clearly that IFN I production by tumor cells is required. Indeed, in these studies, the MAVS or the STING pathway is inactivated. This inactivation impairs IFN I production and also the expression of lots of other genes. Indeed, when the MAVS or the STING pathway are triggered, activated transcription factors such as IRF3 and NF-kB not only induce IFN I production, but also the expression of many other genes with many being ISG (**Figure 2**). In MPM cell lines that have lost IFN I genes, exposition to attenuated measles virus still resulted in the induction of expression of a small subset of genes (21). Among these genes, some may play a role in the inflammation of the microenvironment, such as the chemokines CCL5, CXCL10 and CXCL11, or the type III interferons. Thus, triggering of the IFN I response in tumor cells that have lost IFN I genes may still conserve a certain capacity to inflame the microenvironment. By studying patients with IFN I genes HD tumors, we would better understand the IFN I contribution of tumor cells versus non-malignant cells in cancer development and therapies. We would also better define the contribution of IFN I versus other cytokines/chemokines induced by the triggering of the MAVS or the STING pathway.

IFN I genes HD may also be interesting for new cancer therapies such as antitumor virotherapy using oncolytic replicative viruses. We studied the replication and oncolytic activity of the attenuated Schwarz strain of measles virus (MV) on 22 human MPM cell lines and four types of healthy cell (fibroblasts, mesothelial, endothelial and lung epithelial cells) (69). We found that the healthy cells and seven MPM cell lines were resistant to MV replication due to a protective functional IFN I response. The 15 others MPM cell lines were permissive to MV replication and lysis due to a defective IFN I response. Among these 15 cell lines, 11 were unable to produce IFN I when exposed to MV. We showed later that eight of these 11 cell lines have lost both copies of the IFN I genes (21). The three others cells line have at least one copy of IFN I genes but are not able to produce IFN I in response to the virus suggesting another type of defects of the IFN I response in these MPM cells lines (69). These 11 MV-sensitive MPM cell lines that are unable to produce IFN I in response to MV become MV-resistant if exposed to exogenous IFN I, suggesting that IFNAR signaling is functional in these cell lines. The four other MV-sensitive MPM cell lines were able to produce IFN I in response to MV, but unable to control viral replication suggesting a defect of the IFN I response in IFNAR signaling. This defective IFNAR signaling has been previously reported in tumor cells of some patients with MPM (70). It has been associated to mark decrease of IFNAR, IRF9 and PKR expression and to tumor sensitivity to an oncolytic vesicular stomatitis virus. These studies illustrate the diversity of defects found in the IFN I response from one patients to another in MPM. Such converging selection of tumor cells with a deficient IFN I response highlights the tumor suppressive role of this response.

Other questions are still pending regarding IFN I genes HD. These HD are diverse in length (**Figure 2**). For some patients, only a part of IFN I genes are lost and *IFNB1* gene that encodes IFN-β is preserved (**Figure 1**). Consequences of these partial losses are also to define. During tumor development, do these HD appear concomitantly to *CDKN2A* HD or do they appear later conferring an additional advantage to the tumor variant that carries them? The best techniques for detecting IFN I genes HD is probably FISH assay that can be performed cost effectively on paraffin-embedded tissue and allow to identify homozygous and hemizygous deletions at the single cell level (71). It can also be performed by polymerase chain reaction-based techniques or whole exome sequencing on tumor biopsies, but large amount of non-malignant cells in the biopsy may hide the deletions.

With the success of cancer immunotherapy and recent advances in understanding the IFN I tumor suppressor role, IFN I genes HD should be studied and taken into account in the monitoring of MPM and NSCLC patients. This would likely lead to new strategies and improvements of immunotherapy.

## Author Contributions

Literature review: MG, CC, and JFF. All authors contributed to the article and approved the submitted version.

## Funding

This work was supported by “La Ligue Régionale Grand Ouest contre le Cancer” (CSIRGO: CD16, CD22, CD44, CD49, CD72, CD79 and CD85), “La Ligue Nationale contre le Cancer”, “L’association ARSMESO44 “, “La fondation ARC”, L’Agence Nationale pour la Recherche (ANR-16-CE18-0016), and “LabEX IGO program supported by the National Research Agency *via* the investment of the future program ANR-11-LABX-0016-01”. TD was supported by a grant from Ligue contre le Cancer.

## Conflict of Interest

The authors declare that the research was conducted in the absence of any commercial or financial relationships that could be construed as a potential conflict of interest.
